# Supplementary material for: MetaRibo-Seq measures translation in microbiomes
Source: Nat Commun. 2020 Jun 29;11:3268. doi: 10.1038/s41467-020-17081-z (PMC7324362; doi:10.1038/s41467-020-17081-z)
Supplement: Supplementary file 10 — Supplementary Data 7 [file 41467_2020_17081_MOESM10_ESM.zip › File2/Confidence_High_Taxonomy/207916_out.krona.html]

Javascript must be enabled to view this page.

members
magnitude
magnitudeUnassigned
count
unassigned
taxon
rank

207916\_out

3

2
superkingdom
3

1239
phylum
3

3
186801
class

order
186802
3

family
186803
3

genus
572511
3

species
418240

SRS023526\_contig\_number\_4586SRS1041091\_contig\_number\_20282SRS148159\_contig\_number\_58337
3
